# Revisiting the influence of pH on ^1^J_C_*_α_*_H_ and chemical shifts of glycine and alanine short oligopeptides

**DOI:** 10.1098/rsos.230942

**Published:** 2023-10-04

**Authors:** A. A. Shahkhatuni, A. G. Shahkhatuni

**Affiliations:** Scientific Technological Center of Organic and Pharmaceutical Chemistry of NAS RA, Yerevan, Armenia

**Keywords:** spin–spin coupling constants, amino acids, ionic state, acidity, oligoglycines, oligoalanines

## Abstract

The pH dependence of several NMR parameters of glycine and alanine short oligopeptides has been reported previously in different studies. Here we have thoroughly examined, summarized and demonstrated the dependences of ^1^H, ^13^C and ^15^N chemical shifts and protonation states of amino acids using two-dimensional NMR experiments. Nevertheless, ^1^J_C_*_α_*_H_ one bond spin–spin coupling constants are more informative and convenient for determination of the position and protonation state of glycine and alanine residue in the oligopeptide chain. In particular, for various oligopeptides (up to six residues), it was shown that the pH dependence of ^1^J_C_*_α_*_H_ of N-terminal glycine and alanine residues is larger than that of C-terminal groups, and in backbone residues, it is not influenced by pH and only slightly depends on the position of the amino acid residue in the chain.

## 1. Introduction

Amino acids and short sequence oligopeptides, besides serving as building blocks for proteins, are biologically very active compounds, modulating various molecular and cellular processes [1–9]. Amino acids are astonishing molecules, which can simultaneously manifest themselves in various forms with different properties. Particularly, they can exist in one of four tautomeric forms (anionic, cationic, neutral or zwitterionic) in aqueous solutions, and make transitions among them. The relative amount of each form in the solution depends on various parameters of the medium: temperature, acidity and the presence of other compounds. The main factor is the pH of the solution, which can be easily changed by the addition of an acid or a base. Each tautomeric form is characterized by its very different electrostatics, and the existence of measurable parameters sensitive to electric fields is important for distinguishing the state of amino acid in solution.

It is known that NMR parameters are highly sensitive to external and internal electric fields [10–13]. The electrostatic effects on the CC, CH, CR and CN bonds of amino acids depend on the ionic state and are significantly different, which should be reflected in corresponding NMR parameters and allow differentiation of the ionic state of amino acid. Indeed, NMR spectroscopy has been long regarded as a convenient, fast and accurate method for investigation of the protonation state and determination of the pK_a_ of tautomeric compounds [14,15].

Glycine and its oligopeptides can often be found in the backbone of various biomolecules, as well as in their terminal parts. They are the simplest objects among short oligopeptides, thus, are very attractive for investigation by experimental and computer simulation methods. The tautomerization and the pH dependence of glycine and glycine containing oligopeptides were widely investigated by various theoretical [16–22] and experimental (capillary electrophoresis, potentiometry, IR and UV spectroscopy, THz spectroscopy, EPR spectroscopy, etc.) methods [23,24], including NMR spectroscopy [25–31]. Previously, it was shown that chemical shifts [32] and ^1^J_C_*_α_*_H_ one bond spin–spin coupling constants (SSCC) [33] of amino acid residues in various (backbone, C- or N-terminal) positions in peptides are different. However, these studies are limited, exploring only one of a few possible NMR parameters in di- or tripeptides.

Here we undertook the systematic study of the pH dependencies of chemical shifts and SSCCs of glycine and alanine residues in oligopeptides with various chain lengths (up to six residues). The goal was to find, summarize and clearly show the characteristic distinctions in the NMR parameters, which could determine the state and the position of glycine and alanine residues in the oligopeptide chain.

## 2. Material and methods

Oligopeptides with natural abundance of ^13^C and ^15^N were mostly purchased from Sigma Aldrich. D_2_O and enriched glycine (2–^13^C, 99%; ^15^N, 98%+) were purchased from Cambridge Isotope Laboratories. All alanine and allylglycine residues in oligopeptides have L conformation.

**Figure 1.**
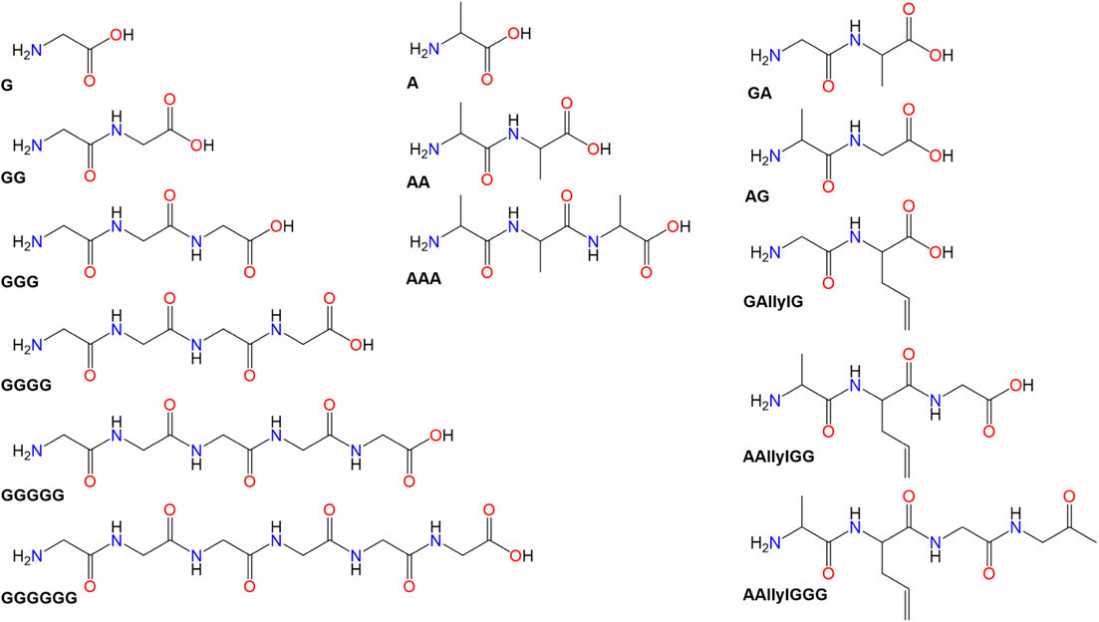
Structures of studied glycine and alanine short oligopeptides, where G, glycine; A, alanine; AllylG, allylglycine.

The structures and used abbreviations of all studied oligopeptides are given in figure 1.

Initial composition of all samples with GA, AG, GG, AA and GGG is the same (1 mM in 800 µl of D_2_O). The pH of the medium was changed by addition of NaOH or CF_3_COOD containing solutions. The composition of samples with longer oligopeptides varies because of low solubility; the longer the peptide the less soluble it is [34–36]. Moreover, peptides with longer chains dissociate at strongly basic pH, and hexaglycine dissociates even at neutral pH [37]. At strongly basic pH the peptide bonds break in oligopeptides with the number of residues *n* higher than three (*n* > 3), forming shorter oligopeptides. For instance, the appearance of NMR spectral signals of GG and G can be clearly seen in the case of oligoglycines. Thus, it was not possible to determine NMR parameters of oligopeptides with *n* > 3 in strongly basic solutions.

The pH measurements were done using Milwaukee Mi-150 pH-meter and special glass electrodes designed for NMR tubes purchased from Hanna Instruments, Inc. The pH values were measured directly in the NMR tube after recording the corresponding NMR spectra. The pH-meter was calibrated in H_2_O, and the direct reading pH values in D_2_O solution were converted to the pD values using the following equation: pD = pH + 0.4 [38].

NMR spectra were acquired at 303 K on a 400 MHz Bruker AVANCE NEO spectrometer equipped with a temperature controlled Smart probe and Varian Mercury 300 VX spectrometer equipped with standard broadband probe and variable temperature unit.

A large set of ^1^H, ^13^C{^1^H} and ^13^C spectra were recorded and ^1^H, ^13^C chemical shifts and ^1^J_CH_ spin–spin coupling constants were obtained. The ^1^J_C_*_α_*_H_ couplings were determined from ^13^C satellite lines in ^1^H spectra and/or from ^13^C non-decoupled spectra. Furthermore, the large set of various two-dimensional NMR spectra were recorded for unambiguous assignments and illustrations for ^1^H, ^13^C, ^15^N chemical shift dependences on pH.

For most of the samples, the number of transients in ^1^H spectra was at least 64 to allow accurate determination of satellite lines. The linewidths for most of the signals were about 1 Hz, and digital resolution is less than 0.1 Hz. For ^13^C spectra, the number of transients was minimum 512. For the compounds with poor solubility, the number of scans was increased as necessary to achieve good signal-to-noise ratio. For two-dimensional spectra, the number of transients varied from 16 to 64. As a reference, the signal of water was taken in ^1^H spectra, and the lock signal was used for referencing other nuclei.

Methylene protons in CH_2_ group of glycine in AG, GA, GAllylG, AAllylGG, AAllylGGG oligopeptides often give an AB type ^1^H spectrum (see electronic supplementary material [39]). However, ^1^J_C_*_α_*_H_ is the same for both A and B nuclei.

The spectra were processed with MestreNova. A large set of experimental spectra of studied oligopeptides at neutral, high and low pH values are given in the electronic supplementary material [39].

## 3. Results and discussion

Employing NMR spectroscopy for titration offers a significant benefit: the capability to distinguish and simultaneously albeit independently monitor all protonated groups.

**Figure 2.**
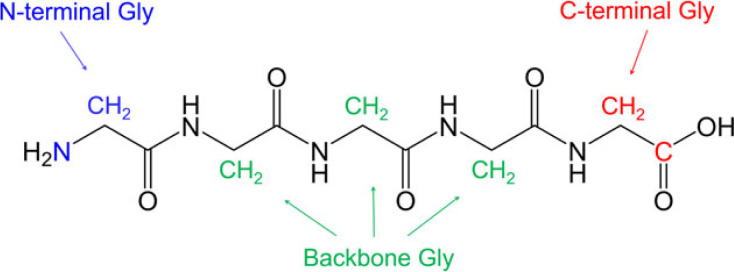
The differentiation of terminal and backbone glycine residues.

Figure 2 illustrates the colour-coded differentiation among N-terminal, C-terminal and backbone glycine residues using the example of pentaglycine. This colour scheme will be consistently used throughout the manuscript.

The positions of glycine and alanine residues in the studied oligopeptides vary (figure 1). For instance, glycine is located either on N-terminal, C-terminal, backbone position or a combination of these.

The most interesting and at the same time the simplest oligopeptide for our studies is triglycine, since it contains only one glycine residue in each possible position. Moreover, triglycine is comparably stable at high pH values, enabling us to study its NMR parameters in a wide pH range.

**Figure 3.**
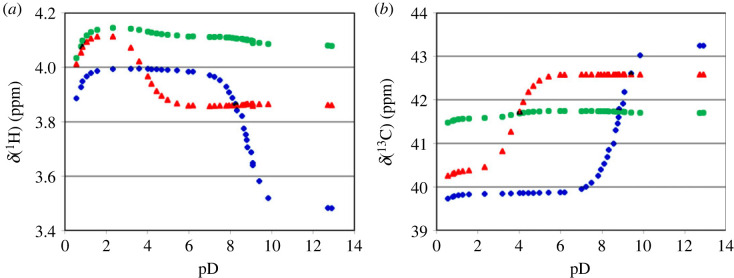
The titration curves of chemical shifts for GGG: GGG (blue diamonds), GGG (red triangles), GGG (green circles).

Figure 3 shows the titration curves of ^1^H and ^13^C chemical shifts of CH_2_ group for all three glycine residues in triglycine. The actual pH of the solution, denoted as pD, was corrected as explained in Material and methods.

**Figure 4.**
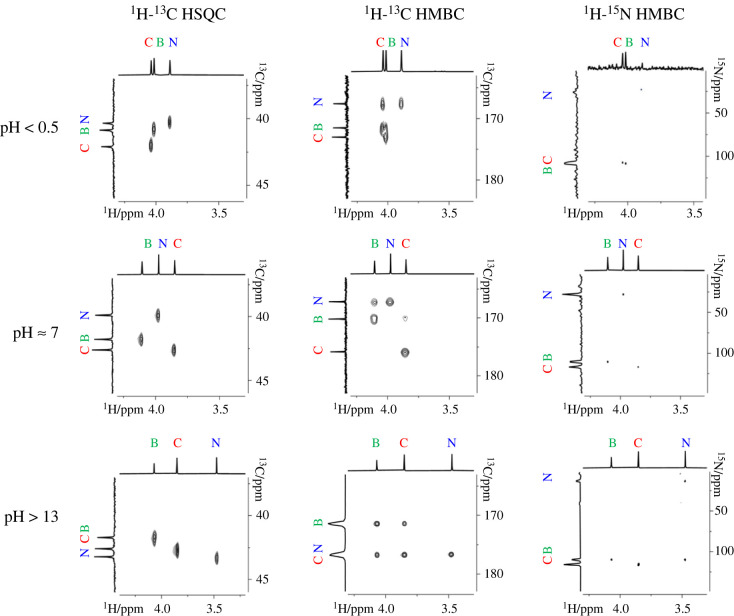
The pD dependence of ^1^H, ^13^C and ^15^N chemical shifts of triglycine shown through two-dimensional correlation NMR spectra. (C, N and B letters denote corresponding nuclei of glycine residues in C-, N- terminal and backbone positions).

In figure 4, two-dimensional correlation spectra demonstrate how pD of the solution affects chemical shifts of ^1^H and ^13^C of CH_2_ group (^1^H-^13^C HSQC), ^13^C of carboxylic group (^1^H-^13^C HMBC) and ^15^N of amine group (^1^H-^15^N HMBC). As expected, the least susceptible to the acidity of the solution are the chemical shifts of nuclei of backbone residue, and the most affected ones are the chemical shifts of N-terminal residue.

^15^N chemical shift of N-terminal residue can always be easily assigned due to its striking difference from chemical shifts of other residues in the peptide chain (figure 4). However, assignments of other chemical shifts require additional two-dimensional NMR experiments, since depending on the pH, the order of the signals of various residues changes (figure 3). Moreover, values of chemical shifts of various short oligoglycines differ from each other, precluding generalizations.

**Figure 5.**
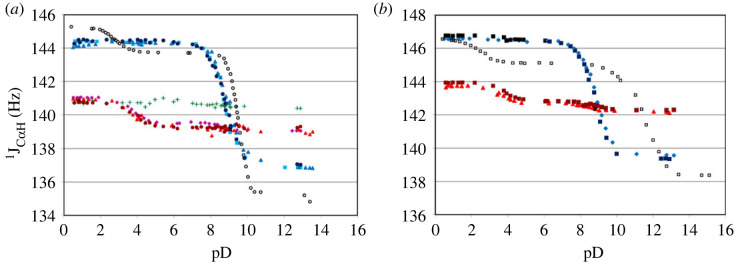
pD dependence of ^1^J_C_*_α_*_H_ for (*a*) glycine residue in N-terminal positions in GA (light blue squares), GG (blue triangles), GGG (dark blue circles); in C-terminal positions in AG (purple diamonds), GG (red triangles), GGG (maroon circles); in backbone position in GGG (plus symbols); and for (*b*) alanine residue in N-terminal positions in AG (blue diamonds), AA (dark blue squares); in C-terminal positions in GA (red triangles), AA (maroon squares). ^1^J_C_*_α_*_H_ of amino acids glycine and alanine are denoted with open circle and square.

To this end, the use of SSCC make results much simpler, more predictable and practical for use, as shown for triglycine [33]. In figure 5, we have constructed the similar ^1^J_C_*_α_*_H_ titration curves for a set of glycine and alanine containing short oligopeptides. The titration curves of glycine and alanine residues in the terminal positions are shown in shades of blue and red, while in the backbone position of triglycine they are shown in green. The most satisfying result is the fact that the titration curves for N-terminal and C-terminal residues are almost the same for all studied oligopeptides. Moreover, there is a clear difference between ^1^J_C_*_α_*_H_ versus pD curves and values at highly acidic, basic and neutral pD of C-, N-terminal and backbone residues. For instance, for the CH_2_ group of N-terminal glycine residue the ^1^J_C_*_α_*_H_ varies from 136.8 to 144.3 Hz, for C-terminal–from 138.9 to 141.0 Hz, and for backbone residue there are no significant changes of ^1^J_C_*_α_*_H_ throughout the whole pD region (from 0.5 to 14). As with chemical shifts, here as well, the residue on the N-terminal exhibits the most pronounced sensitivity to pD changes. The analogous distinctive features of titration curves are observed for ^1^J_C_*_α_*_H_ in alanine (figure 5*b*). Furthermore, the similar pD dependence was found for ^1^J_CH_ of methyl group in alanine residue, albeit the difference in the values in strongly acidic and basic solutions for N-terminal residue is about 3 Hz.

As expected, the dependence of NMR parameters on pH is intermittent, and there are pH ranges, where the parameters do not change, for instance, 5–7 pH range. On one hand, it means that NMR parameters are not that subtle as probes and are suitable only for rough estimation of the acidity of the solution. On the other hand, those ‘static' ranges allow clear and unambiguous determination of the state and the position of the amino acid residue.

**Figure 6.**
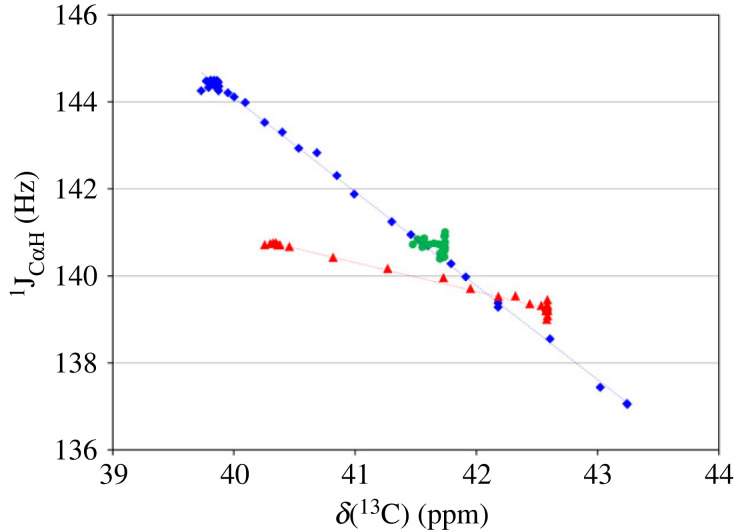
Linear dependence of ^1^J_C_*_α_*_H_ from ^13^C chemical shifts of CH_2_ groups of glycine residues in GGG: GGG (blue diamonds), GGG (red triangles), GGG (green circles).

The changes of *δ*(^13^C) and ^1^J_C_*_α_*_H_ for both terminal residues are linearly dependent (figure 6), which means they are influenced by the same parameter, most likely, the effective charge at the corresponding site. The dependence for N-terminal residue, measured by the slopes of trendlines, is three times more sensitive.

The titration curves for glycine and alanine amino acids are also shown in figure 5 for comparison. They mimick and combine peculiarities of both curves for N- and C-terminal residues although with different starting values. It can be attributed to the nature of amino acid, which can exist in cationic or anionic state depending on the pH of solution. The variations in ^1^J_C_*_α_*_H_ values are more than 10 Hz. Moreover, studies of ^13^C and ^15^N enriched glycine showed that other SSCC are also susceptible to pH. In particular, ^1^J_CC_ changes from 59.5 to 52.1 Hz, ^1^J_CN_ changes from 7.5 to 4.5 Hz at strongly acidic and strongly basic pH, correspondingly.

Study of a larger set of glycine containing short oligopeptides revealed the same tendency; the value of ^1^J_C_*_α_*_H_ is pH dependent and characteristic for the determination of the position of glycine residue in the backbone.

The solutions we have studied are monomolecular, since the number of glycine or alanine residues in our studied oligopeptides (up to six residues) is small to form any elements of secondary structure, and used concentrations are low for triggering aggregation processes. Unfortunately, alongside poor water solubility, longer oligopeptides (starting from four residues) dissociate at high pH values, preventing registration of SSCC values in strongly basic environment.

**Figure 7.**
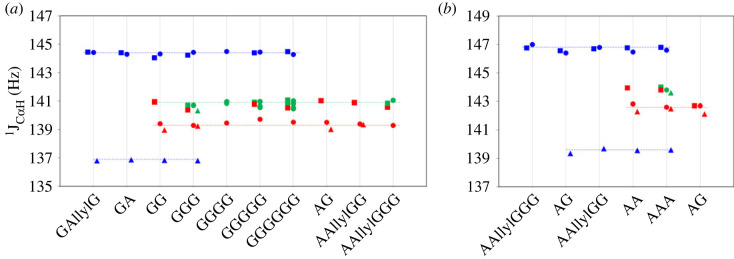
^1^J_C_*_α_*_H_ for N-terminal (blue), C-terminal (red) and backbone (green) (*a*) glycine and (*b*) alanine residues for various short oligopeptides in neat solution (circles), strongly acidic (squares) and strongly basic (triangles) solutions. The values for AAA were taken from [33].

^1^J_C_*_α_*_H_ values of backbone residues of oligopeptides are very close to each other and range from about 140.5 to 141.2 Hz, and are essentially the same at both neutral and highly acidic pH. As stated above, due to the dissociation of oligopeptides in basic media, we could only obtain values for triglycine, which are the same for the whole studied pH range.

In figure 7*a*, the values of ^1^J_C_*_α_*_H_ of CH_2_ group for different glycine and alanine residues for a set of short oligopeptides (up to six residues) in media with various acidity are presented. For all studied oligopeptides, the value of ^1^J_C_*_α_*_H_ in N-terminal residue is almost the same and equals 144.4 ± 0.2 Hz in cationic state (strongly acidic solution, pH < 1) and neat solution (pH ≍ 6–7), and 136.9 ± 0.2 Hz in anionic state (strongly basic solution, pH > 13). On the other hand, ^1^J_C_*_α_*_H_ for all C-terminal glycine residues has the same value of 140.7 ± 0.2 Hz in cationic state, and 139.0 ± 0.2 Hz in anionic state and at neutral pH. Overall, the range of values of ^1^J_C_*_α_*_H_ in different ionic forms for N-terminal glycine residues (about 9 Hz spread) is greater than for C-terminal (about 2 Hz spread).

The similar behaviour was observed for ^1^J_C_*_α_*_H_ of CH group of alanine containing short oligopeptides, although for a smaller set of oligopeptides (figure 7*b*). The ^1^J_C_*_α_*_H_ values differ significantly for N- and C-terminal alanine residues and are almost the same for all studied short oligopeptides. In particular, the values for N-terminal residue are 146.7 ± 0.2 Hz at strongly acidic and neutral pH, and 139.6 ± 0.2 Hz at strongly basic pH.

Thus, the ^1^J_C_*_α_*_H_ values of N-terminal, C-terminal and backbone glycine and alanine residues in oligopeptides are very different, enabling the determination of the position of amino acid residue in peptide chain.

## 4. Conclusion

NMR parameters are useful indicators for determination of the protonation state of terminal glycine and alanine residues and their position in the oligopeptide chain. Chemical shifts differ significantly depending on the acidity of the solution and the position of the glycine or alanine residue in the oligopeptide chain. However, the pH dependence of ^1^J_C_*_α_*_H_ is more unambiguous and general, and can serve for differentiation of the state of amino acid residue and its terminal position in the oligopeptide chain.

In particular,

— ^1^J_C_*_α_*_H_ of all N-terminal glycine and alanine residues have values 144.4 ± 0.2 Hz and 146.7 ± 0.2 Hz in cationic state, 136.9 ± 0.2 Hz and 139.6 ± 0.2 Hz in anionic state for all studied oligopeptides.
— ^1^J_C_*_α_*_H_ of all C-terminal glycine and alanine residues have the same values of 140.7 ± 0.2 Hz and 143.8 ± 0.2 Hz in cationic states, and 139.0 ± 0.2 Hz and 142.2 ± 0.1 Hz in anionic state for all studied oligopeptides.
— The range of values of ^1^J_C_*_α_*_H_ in different ionic states of N-terminal glycine and alanine residues is greater than that of C-terminal.

## Data Availability

The data are provided in electronic supplementary material [39].
